# Fasting to enhance Cancer treatment in models: the next steps

**DOI:** 10.1186/s12929-020-00651-0

**Published:** 2020-05-05

**Authors:** Jing Zhang, Yanlin Deng, Bee Luan Khoo

**Affiliations:** Department of Biomedical Engineering, City University of Hong Kong, 83 Tat Chee Avenue, Kowloon Tong, Hong Kong

**Keywords:** Cancer metabolism, Tumor progression, Short-term fasting, Chemotherapy, Tumor models

## Abstract

Short-term fasting (STF) is a technique to reduce nutrient intake for a specific period. Since metabolism plays a pivotal role in tumor progression, it can be hypothesized that STF can improve the efficacy of chemotherapy. Recent studies have demonstrated the efficacy of STF in cell and animal tumor models. However, large-scale clinical trials must be conducted to verify the safety and effectiveness of these diets. In this review, we re-examine the concept of how metabolism affects pathophysiological pathways. Next, we provided a comprehensive discussion of the specific mechanisms of STF on tumor progression, derived through studies carried out with tumor models. There are currently at least four active clinical trials on fasting and cancer treatment. Based on these studies, we highlight the potential caveats of fasting in clinical applications, including the onset of metabolic syndrome and other metabolic complications during chemotherapy, with a particular focus on the regulation of the epithelial to mesenchymal pathway and cancer heterogeneity. We further discuss the advantages and disadvantages of the current state-of-art tumor models for assessing the impact of STF on cancer treatment. Finally, we explored upcoming fasting strategies that could complement existing chemotherapy and immunotherapy strategies to enable personalized medicine. Overall, these studies have the potential for breakthroughs in cancer management.

## Introduction

Dietary and lifestyle habits are the two key factors that escalate cancer risk. The onset of specific cancer types, such as colorectal cancer and breast cancer, has shown a stronger correlation with nutritional habits [1, 2]. Among these dietary habits, obesity is the leading cancer risk factor, especially for endometrial cancer. Variations of certain nutrient levels, such as vitamins, trace elements, and dietary fat, can also generate a cancer-promoting environment. Although the link between obesity and cancer is well documented, many are still oblivious to the impact of a poor diet and a sedentary lifestyle on the elevated risk of cancer. In the United States, obesity accounts for nearly 14–20% of all cancer-related mortality. Many current studies have focused on the role of nutrients in the treatment of certain diseases, such as cardiovascular disease, diabetes and cancer.

Chemotherapy is a systemic therapy that can cause severe side effects such as anemia, nausea, hair loss, organ damage, and neutropenic enterocolitis [3]. Although this treatment is most successful for some tumors (such as testicular cancer and specific leukemia subtypes), it is not sufficient for other cancer types [3, 4].

Recent studies suggested that a combination of fasting and chemotherapy can improve the efficacy of treatment [5]. Based on the research, several nutrient-related therapeutic interventions have been proposed, including the concurrent application of fasting during chemotherapy treatment. For example, compared with either strategy alone, fasting combined chemotherapy is more effective in delaying the progression of various tumors and reducing the number of organs affected by melanoma metastases [6]. Preclinical studies based on cell and animal models have also shown that fasting can enhance the efficacy of multiple chemotherapeutic drugs for cancer and protect rodents from the toxic effects of chemotherapy. These studies on breast cancer, neuroblastoma, and colorectal cancer mouse models show that fasting can extend overall survival by protecting healthy cells from chemotherapy while effectively inhibiting tumor progression [6].

## Fasting strategies and clinical trials

The importance of metabolism in cancer progression indicates that dietary habit is a huge factor in cancer outcomes [7]. Therefore, various fasting techniques have been developed to exploit the nutrient dependency of cancer. Most fasting durations are usually short-termed, ranging from 24 to 72 h. The following sections outline the types of fasting strategies and the preliminary observations obtained from ongoing clinical trials.

### Types of fasting strategies

Fasting and other dietary interventions are categorised based on the duration of fasting, as shown in Table 1.

**Table 1:** Categories of fasting and other diet intervention methods

Category	Diet intervention	Duration	Ref examples
**Short term fasting (STF)**	0 cal intake	24 h to 5 days	[8–12]
**Dietary restriction (DR)**	20% ~ 40% reduction of calorie intake	Acute: 2 weeks; Moderate: 3–19 weeks; Long: at least 20 weeks	[13, 14]
**Intermittent fasting (IF)**	70% energy restriction	Every other day	[15]
	500–700 cal intake	Two consecutive days per week	
**Fasting-mimicking diet (FMD)**	Fasting for two days (Day 1 provided at about 50% of normal daily intake, day 2–4 provided at about 10% of normal daily intake) followed by ten days *ad libitum* refeeding	Months	[16, 17]
**Periodic Fasting and Refeeding Cycles (PFRC)**	1–2 days STF and 5–6 days *ad libitum* refeeding	Months	[18]

The most common strategy is short-term fasting (STF), a technique that involves zero-calorie intake and ranges from 24 to 72 h. Some reports state that STF may be carried out for extended periods (such as five days) [8].

In contrast, dietary restriction (DR) is a technique to reduce calorie intake by 20 to 40% over a long period [13]. It has been found that DR techniques were beneficial for the prevention and treatment of cancer [19]. Studies demonstrating the impact of DR have shown that methionine (MET) dependence is a significant metabolic feature of tumors [20] and that MET restriction (MR) has been demonstrated to generate tumor-inhibiting effects and promote chemotherapeutic sensitivity [21]. The most common models of DR studies involve invertebrates such as *Saccharomyces cerevisiae*, *C. elegans*, *E. coli,* and *Drosophila*. Studies had shown that when these invertebrate models were subjected to DR, their lifespans were prolonged [22]. These observations are supported by other primate models such as the rhesus monkeys, where there was reduced aging and delay in morbidity and mortality [23, 24]. However, the potential utility of DR in clinical settings is limited because such strategies not only require significant changes to dietary patterns but also inevitably cause chronic weight loss, especially in patients undergoing cancer chemotherapy [6]. Models subjected to DR have also shown delayed wound healing and impaired immune function [13, 14, 25].

Intermittent fasting (IF) is another way to limit nutrient intake. IF techniques require a 70% energy restriction every other day, and patients consume only 500–700 cal two consecutive days a week [15]. The IF strategy has been associated with a reduction of cancer onset and found to prolong lifespans, without the presence of chronic weight loss [13, 26]. Periodic fasting (PF) is a modification of the IF strategy, in which patients under PF are subjected to fasting or fasting-mimicking diet (FMD) (see Periodic Fasting and Refeeding Cycles (PFRC)) between 2 to 21 days [16].

### Observations from clinical trials

Currently, results from clinical trials support the implementation of fasting to enhance chemotherapy. STF combined with chemotherapy (24 h before and after chemotherapy) was found to reduce the hematological toxicity of neoadjuvant docetaxel/doxorubicin/cyclophosphamide treatment (TAC) with epidermal growth factor receptor 2 (HER2)-negative early breast cancer patients and speed up recovery of DNA damage in peripheral blood mononuclear cells (PBMCs) [27]. Another study showed that almost no side effects, such as nausea, vomiting, and mucositis due to chemotherapy, were observed. The study involved ten patients with various types of early or advanced cancers, including prostate cancer, breast cancer, and esophageal adenocarcinoma who received carboplatin, docetaxel, paclitaxel or gemcitabine treatment at least 48 h before or at least 5 h after chemotherapy [8].

A stochastic crossover trial also evaluated the impact of STF (36 h before fasting and 24 h after chemotherapy) on the living quality of patients with ovarian cancer and breast cancer treated with chemotherapy. The results show that STF is well tolerated during chemotherapy, improves the quality of life, and reduces fatigue in patients with primary and advanced gynecological cancers (breast and ovarian cancer) during chemotherapy [28, 29]. In another study involving urothelial, ovarian, and breast cancer patients undergoing platinum-based chemotherapy, fasting for 72 h during chemotherapy could reduce DNA damage of leukocytes [30]. These trials have also suggested that fasting can confer protection to host tissues against chemotherapy damage [30].

Potential issues of fasting that require further investigation include metabolic complications such as malnutrition and sarcopenic obesity, as demonstrated in a study on prostate cancer [31]. The prevalence of malnutrition and sarcopenia may be widespread (up to 80%) in cancer patients and is correlated with the stage and site of the tumor [32]. Therefore, fasting procedures in clinical settings are not well defined, and such treatment procedures will be limited to specific patient subgroups (see Potential caveats of implementing fasting strategies).

## Nutrients and Cancer progression

Regulation of nutritional levels is a delicate balance between the maintenance of fundamental physiological functions and the elevation of pathological disease risk. In the following sections, we summarize the physiological changes after fasting and the key mechanisms by which nutrients affect cancer progression.

### The first physiological changes upon fasting

**Fig. 1:**
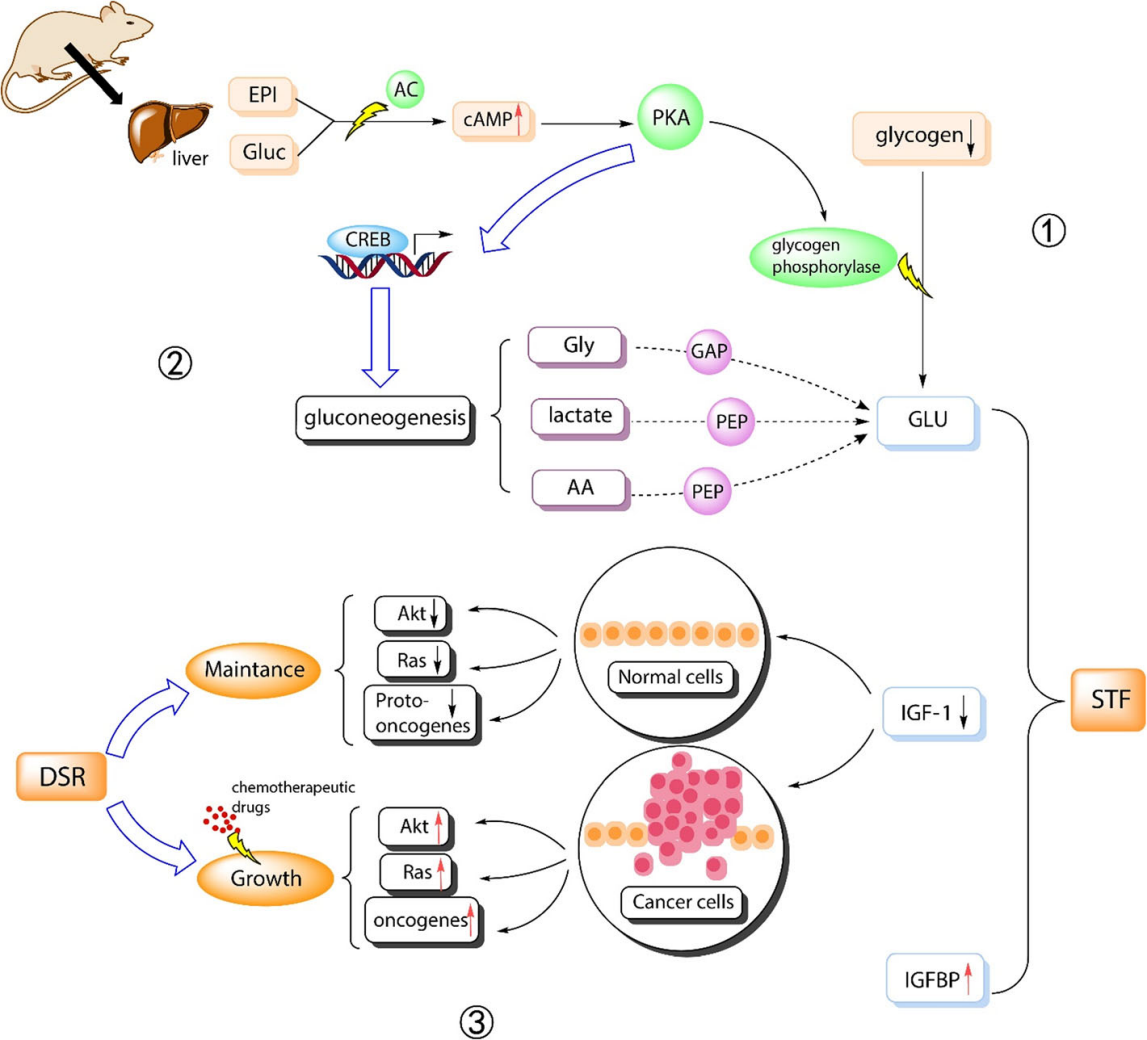
Key findings from existing tumor models on the correlation between nutrients, stress response, and cancer. Schematics of the interconnecting feedback loops between nutrient levels and stress response. Step 1: The cascade of kinase action. Fasting triggers elevated levels of glucagon (Gluc) and epinephrine (EPI), which in turn induces a cascade of cyclic adenosine monophosphate (cAMP)-dependent signaling via adenylate cyclase (AC). This reaction then leads to the activation of protein kinase A (PKA) and glycogen phosphorylase, which promotes glycogenolysis. Step 2: Process of gluconeogenesis. Amino acids (AA), lactate, and glycerol (Gly) are substrates of gluconeogenesis [36] and glycerol-phosphoric acid (GAP), and phosphoenolpyruvate acid (PEP) are intermediates of the reaction. In addition to the involvement of certain key enzymes such as pyruvate carboxylase, transcription factors such as cAMP response element-binding protein (CREB) are also involved in the process of gluconeogenesis [35]. Step 3: The onset of DSR - Under fasting conditions, Gluc and EPI initiate the cascade of kinase action that releases glucose from the stored glycogen via the process of glycogenolysis [34]. CREB is critical in coordinating the fasting-mediated activation of gluconeogenesis in the liver [34]. The extreme changes caused by fasting include reduction of IGF-I and glucose (GLU) and an increase in IGFBP

The liver plays a significant role in homeostasis by maintaining blood glucose balance, which is essential for many mammalian cells [33]. Under fasting conditions, the liver can synthesize glucose as an energy source for other tissues, including the brain and muscles [34]. After approximately 30 h of fasting, almost all the stored glycogen in the liver is depleted, and the liver starts to synthesize de novo glucose or gluconeogenesis [34]. In addition to the involvement of certain key enzymes, transcription factors such as cyclic adenosine monophosphate (cAMP) response element-binding protein (CREB) are also involved in the process of gluconeogenesis [35]. CREB is one of the major transcriptional factors that has been shown to induce gluconeogenesis (Fig. 1, steps 1–2) [37]. Protein kinase A (PKA) activation then induces CREB through its serine 133 phosphorylation [38].

### Nutrient levels influence mechanisms that promote Cancer development

Fluctuations in nutritional levels can trigger anti-carcinogenic effects through modulation of the apoptotic pathways. For instance, vitamin C upregulates the expression of TNF-related apoptosis-inducing ligand (TRAIL). TRAIL is a crucial member of the tumor necrosis factor (TNF) receptor superfamily. It regulates tumor cell apoptosis in a p53-independent manner, thereby inducing cancer cell apoptosis [39, 40]. In a recent study involving lung, breast, bladder, and colon cancer models, it was found that γ- tocotrienols (γ-TT) and δ-tocotrienols (δ-TT) generate the most significant anti-carcinogenic effects within the vitamin E group. Specifically, δ-TT induced cell cycle arrest reduced cancer cell metastasis and inhibited the angiogenesis process [41]. Dietary n-3 polyunsaturated fatty acids (n-3 PUFAs) also have anti-inflammatory effects, so they can also prevent cancer [42]. These negative correlations with breast cancer risk have also been confirmed in prospective cohort studies [43].

**Fig. 2:**
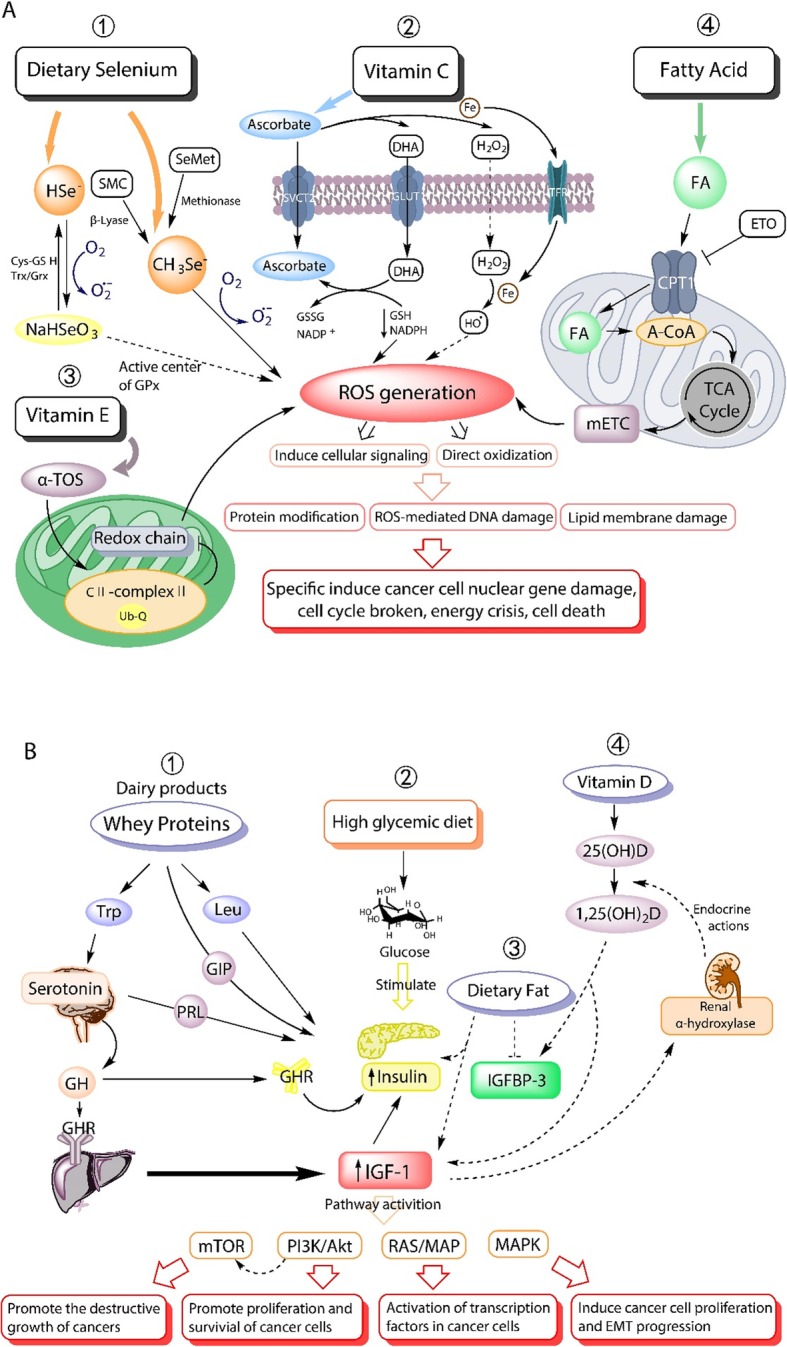
Metabolism affects stress response and cancer progression. **a** The effects of nutrients on cellular stress response through the production of reactive oxygen species (ROS). Step 1: Metabolism of selenium and its impact on ROS generation. Step 2: Oxidation of vitamin C in cells and the process of generating H_2_O_2_ and ROS. Step 3: Mechanism of α-tocopheryl succinate (α-TOS) promoting ROS generation via damaging redox chain in the mitochondria. Step 4: Fatty acid (FA) induced ROS levels by promoting the carboxylic acid (TCA) cycle in the mitochondria. **b** The effects of nutrients on cancer progression via insulin, IGF-1, and IGFBP-3 levels: Step 1: Whey protein increases the levels of insulin and IGF-1 by directly stimulating insulin levels or through growth hormone (GH) and growth hormone receptor (GHR). Step 2: Stimulation from high glycemic diet to insulin generation Step 3: Effects of dietary fat on the circulating levels of insulin, IGF-1, and IGFBP-3. Dietary fat could stimulate insulin levels and circulating IGF-1 levels while inhibiting the expression of IGFBP-3. Step 4: The interactions of vitamin D, IGF-1, and IGFBP-3 can form a positive feedback circuit, and intake of Vitamin D can increase the IGF-1 levels to some extent. The increase of IGF-1 levels activates different signal pathways, which then affects cancer progression

Other nutrient types can also affect redox balance. Redox balance in the tumor environment is dysregulated by high metabolic rate and redox-related enzyme activities. Therefore, reactive oxygen species (ROS) levels are often increased significantly [44, 45]. Selenium is a critical factor in the antioxidant process of cells. The primary dietary metabolites are hydrogen selenide (HSe^−^) and methylselenol (CH_3_Se^−^) (Fig. 2a, step 1). Dietary metabolites of selenium play a vital role as the active center of glutathione peroxidase (GPx). Selenium metabolites also are used as pro-oxidants in the redox cycle with glutathione (GSH) or other components in the Trx/Grx pathways. HSe^−^ and CH_3_Se^−^ can generate superoxide (O_2_^−^) and hydrogen peroxide (H_2_O_2_) through oxidation, further facilitating ROS generation [46–48]. Vitamin C can also increase ROS levels through the oxidation cycle and the production of dehydroascorbic acid (DHA) and H_2_O_2_ (Fig. 2a, step 2) [49].

As the most efficient form of vitamin E, α-tocopheryl succinate (α-TOS) has also been proven to interfere with the ubiquinone (UbQ) binding site of mitochondrial complex II, leading to an impairment of the electron transfer along the redox chain and stimulate ROS production (Fig. 2a, step 3) [50]. Besides, fatty acids derived from dietary fats can also induce ROS production by promoting the carboxylic acid (TCA) cycle in mitochondria (Fig. 2a, step 4) [51]. Overall, high oxidative stress and abnormal ROS-related pathways in cancer cells, especially advanced cancer, are a few of the key mechanisms of anti-cancer techniques targeting metabolism-related pathways [52, 53].

Insulin-like growth factor-1 (IGF-1) is a key component that can specifically interact with IGF binding proteins (IGFBP). IGF-1 deficiency can confer protection against tumor progression [54]. IGF-1 participates in various growth-related pathways by altering protein structure or binding to the cell-matrix to regulate cell growth, differentiation, and nutrient metabolism, thereby influencing tumor cell division or anti-apoptotic pathways [55, 56]. Insulin-like growth factor binding protein-3 (IGFBP-3) is an effective apoptotic factor regulated by p53. As a significant member of the IGFBP family, it can bind to circulating IGF-1 and regulate its mitogenic and anti-apoptotic effects [57, 58]. The growth-inhibiting result of IGF-3 can also be non-IGF-dependent. Inhibition of IGFBP-3 expression occurs in a variety of cancers, such as lung and ovarian cancer, which makes it a potential target for tumor suppressor genes [59].

Whey proteins obtained from dairy products can increase insulin and IGF-1 levels (Fig. 2b, step 1). Tryptophan (Trp) and leucine (Leu) are two amino acids rich in α-lactalbumin and can increase both insulin and IGF-1 levels. Leu can directly stimulate the production of insulin in the pancreas. In contrast, Trp can stimulate the generation of growth hormone (GH) in the hypophysis, in combination with the growth hormone receptor (GHR), thereby increasing the generation of IGF-1 in the liver [60].

### Metabolic pathways that promote Cancer progression

Homeostasis regulation of insulin and IGF-1 levels activate various pathways to mediate cell proliferation and survival, which can promote cancer cell proliferation and invasion or inhibit apoptosis [61]. Clinical studies have shown that high levels of IGF-1 in the circulation could increase the risk of prostate, breast, and colorectal cancer [62–64]. In addition, a hyperglycemic diet has been shown to affect the IGF-related pathways and is positively correlated with cancer progression by directly overstimulating insulin production (Fig. 2b, step 2) [60].

Studies have also shown that excessive intake of dietary fat increases the risk of prostate and pancreatic cancer [65, 66]. Large amounts of dietary fat could also increase circulating IGF-1 levels and reduce IGFBP-3 expression, thereby stimulating the IGF signaling cascade. Stimulating the IGF signaling cascade can promote cancer cell survival (Fig. 2b, step 3).

The correlation between vitamin D levels and IGF-1 levels has also been reported (Fig. 2b, step 4) [67, 68]. Studies have shown that vitamin D promoted the circulating levels of IGF-1 and IGFBP-3 by increasing intestinal calcium absorption, leading to a negative correlation between vitamin D levels and breast cancer risk.

A high-folate diet (vitamin B_9_) could also promote breast cancer growth by activating cancer-related genes to promote DNA replication in tumor cells [69]. Excessive intake of vitamin B_12_ and folic acid could also promote tumor progression [69, 70].

### Effect of fasting on the cancer-related pathways

Fasting strategies aim to promote metabolic pathways that stimulate anti-carcinogenic effects, such as the insulin signaling pathway. The inhibiting effect of fasting towards cancer progression occurs by the blocking of energy intake and nutrients to affect other metabolism-related pathways. Sirtuin 1 (SIRT1) is an NAD^+^-dependent histone deacetylase that negatively regulates liver GH-mediated IGF-1 mRNA production, thereby reducing serum IGF-1 levels [71]. In the fasting state, the activation of AMP-activated protein kinase(AMPK) can promote lipid oxidation and increase the ratio of NAD^+^ / NADH, thereby activating SIRT1 and then inhibiting IGF-1 production [72]. Due to the reduction of IGF-1 during fasting, it has been shown that the IGF-1 binding protein IGFBP-1, which can reduce IGF-1 levels and block the IGF-1 signaling pathway, can increase significantly 11.4 times in 72-h STF [73].

Fasting also results in lower blood glucose levels and lower circulating insulin levels (Fig. 2b, step 2). Since insulin promotes the PI3K/Akt signaling pathway through the insulin receptor (IR) or IGF-1 receptor [74], a decrease of insulin due to fasting can inhibit the PI3K/Akt pathway and disrupt the balance of energy gain and consumption. Inhibition of the PI3K/Akt pathway further limits the target of rapamycin (TOR) complexes 1 (mTORC1) [75] and prevents activation of the mTOR pathway, which increases protein synthesis in tumor cells [9]. PI3K/Akt signaling can also promote glucose metabolism by inhibiting GSK-3β and reduce cell apoptosis by destroying the BCL2-Bad complex to promote cancer progression [76]. In addition to the PI3K / AKT / mTOR integrated pathway, fasting can also inhibit the activity of the RAS/MAP nutrient signaling pathway by reducing IGF-1 levels, inhibiting the activity of transcription factors in tumor cells, and subsequently expressing genes involved in proliferation and cell growth [77, 78].

Also, it has been shown that TRAIL levels in the natural killer (NK) cells of fasting mice can be up-regulated by HSP70 expressed on the surface of tumors, where HSP70 is a heat shock protein (HSP) produced in excess under fasting conditions [79]. TRAIL^+^ NK cells have been shown to attack transformed cells, including tumor cells.

## Current insights on the efficacy of fasting strategies

The following sections detail the latest insights on fasting based on studies of cell and animal models, highlighting the effects of fasting on cancer prevention, enhancing chemotherapy, preventing chemotoxicity, stress response, and metastasis. We will also discuss the potential caveats of fasting-related studies due to cancer stages, types, and tumor heterogeneity.

### The impact of fasting on Cancer progression and therapy

#### Cancer prevention and treatment enhancement

The possibility that fasting can improve the efficacy of standard chemotherapy by complementing treatment or by increasing the tolerance of patients to chemotherapeutics (Table 2) has gained a considerable amount of interest among oncologists [6]. Overall, research shows that fasting can be involved in the prevention and treatment of cancer. Fasting results in extensive changes in growth factor and metabolite levels in cells and animal models, creating an environment that may reduce the ability of cancer cells to adapt and survive, thereby increasing the effectiveness of cancer treatment. Fasting has also been found to promote the regeneration of healthy tissue, which helps prevent harmful and potentially life-threatening side effects of chemotherapy.

**Table 2:** The effects of fasting on tumor progression during chemotherapy. Period = length of fasting; MC = multiple cycles of fasting; Y = yes; N = no.

Period	Model	Cancer type	Cancer prevention	Treatment enhancement	Protective effect	Ref
	**Cell lines**					
24 h	BxPC-3, MiaPaca-2 and Panc-1	Pancreatic		Y		[9]
24 h	SKBR3, BT474, HCT116, HCC827, H3122	Colorectal, breast	Y	Y		[80]
24 h	U251, LN229, and A172	Glioblastoma		Y	Y	[11]
24 h	LN229, SH-SY5Y	Glioma, neuroblastoma		Y	Y	[81]
48 h	CT26	Colon	Y			[10]
	**Animal**					
72 h	Male BALB/c mice	Colon			Y	[12]
24 h	Female Nu/Nu mice	Pancreatic	Y			[9]
48 h (MC)	Female BALB/c mice	Colon	Y			[10]
72 h	LID mice	Melanoma	Y		Y	[73]
48 h (MC)	BALB/c and C57BL/6 mice	Neuroblastoma, breast, ovarian		Y	Y	[6]
48 h (MC)	Female BALB/c athymic mice	Colorectal, breast	Y	Y		[80]
24–48 h (MC)	Male C57BL/6 N mice	Glioblastoma		Y	Y	[11]
24 h	Canine	Lymphoma			Y	[82]

Studies in cell and animal models have shown that fasting can trigger autophagy [83, 84], complementing the role of anti-cancer drugs that target the apoptotic pathway. Autophagy plays a variety of roles in cancer and is a self-regulating mechanism that allows orderly degradation and recycling of cellular components [85–87]. Autophagy is mediated by tumor suppressors such as PTEN and TSC2. Oncogenic signals, such as PI3K and Akt, can inhibit autophagy [13]. As an adaptive response of cells, autophagy can redistribute energy from growth to protection in healthy cells, degrade damaged proteins and organelles to produce amino acids as alternative energy sources [83, 88, 89]. Various forms of stress can also stimulate it, including a lack of nutrients or growth factors, hypoxia, production of ROS, and DNA damage [83]. In cancer, metabolic changes are a hallmark of cancer, and increased autophagy is usually the result of ammonia-induced mitochondrial dysfunction mediated by the Warburg effect. The Warburg effect suggests that cancer cells are known to be more dependent on glycolysis than oxidative phosphorylation [84, 90]. In a study on colon cancer, STF up-regulated oxidative phosphorylation in cancer cells and down-regulated anaerobic glycolysis, leading to an ‘anti-Warburg effect.’ This ‘anti-Warburg effect’ resulted in oxidative stress and induced apoptosis of tumor cells [10]. Glutamine is another metabolic substrate that has a high conversion rate in tumor cells [91]. The catabolism of glutamine can protect tumor cells from chemical toxicity. Therefore, STF can increase the sensitivity of cancer cells to chemotherapy via glutamine catabolism by affecting glutaminase and glutamine transporter levels and subsequently preventing amino acid biosynthesis [10]. The reduced availability of nutrients during fasting also makes cancer cells more susceptible to chemotherapy [29].

Studies also found that the presence of congenital IGF-1 deficiency, equivalent to reducing IGF-1 through fasting (see Nutrient Levels Influence Mechanisms that Promote Cancer Development), could confer protection against tumor progression [54]. These results obtained from neuroblastoma, breast, and ovarian tumor models indicate that tumor growth can be inhibited by 48-h fasting under doxorubicin (DXR) and cyclophosphamide (CP) treatment (Table 2) [6].

Fasting also enhances the effectiveness of tyrosine kinase inhibitors (TKIs), a therapy commonly utilized in several types of cancer, by inhibiting mitogen-activated protein kinase (MAPK) signaling pathways and inhibit cancer cell growth(Fig. 2b) [80].

#### Protective effect against Chemotoxicity

Oxidative stress is a result of free radicals and other ROS produced during chemotherapy. When exposed to chemotherapy drugs, it reduces the rate of cell proliferation and produces side effects such as gastrointestinal toxicity and mutagenesis [92]. ROS production may also lead to renal toxicity (induced by cisplatin), cardiotoxicity (induced by doxorubicin), and pulmonary fibrosis (induced by bleomycin) [92]. ROS can also lead to DNA damage and modifications [93].

Due to the different metabolic pathways of healthy and cancer cells [6], fasting has protective effects on oxidative stress and side effects of chemotherapy in healthy cells. This process is termed as the differential stress resistance (DSR) (see Resistance and Stress Response) and has been widely demonstrated in animal models (Table 2) [6, 11, 81]. For example, a 48 h period of fasting before chemotherapy treatment was found to mitigate acute cardiotoxicity in mice. Acute cardiotoxicity is a side effect of irreversible degenerative cardiomyopathy and congestive heart failure caused by doxorubicin [94]. Significant reductions in the incidence of chemically induced nausea and vomiting detected under fasting have also been observed in other studies [82]. Fasting was also found to reduce the frequency of side effects from irinotecan, a drug commonly used to treat colon cancer and small cell lung cancer, without the interference of its anti-tumor efficacy [12]. Without fasting, the use of irinotecan is limited by many side effects, such as neutropenia and diarrhea. Overall, results from cell and animal models suggest that fasting reduces chemotherapy-induced toxicity and differential stress sensitization (DSS). DSS is a phenomenon in which cancer cells are more sensitive to chemotherapy than healthy cells in extreme environments [22]. For example, genes associated with the insulin signaling adaptor (*Irs2*) were down-regulated in healthy breast epithelial cells while they were upregulated or unaffected in breast cancer cells [6]. Similarly, results were observed in the expression of genes associated with the elongation factor 1γ (*Eef1g*), associated with oncogenic transformation [95]. Specifically, in healthy cells, the expression of *Eef1g* was repressed or minimally affected, but it was significantly increased in breast cancer cells [6].

#### Resistance and stress response

With the appreciation of the impact that nutrients have on the redox balance, it is not surprising that fasting is associated with stress resistance, most notably oxidative stress. In bacteria cultures, *E. coli* showed enhanced resistance to heat or H_2_O_2_ challenge when cultured in the absence of glucose or nitrogen. The heat and chemical resistance exhibited by bacteria in the absence of nutrients depended on the period of starvation [96]. Fasting could also increase resistance to anoxia (reoxygenation) stress in the Drosophila model [97]. In the canine model, the reduction of glutamine also prevents the onset of lipolysis and ketogenic action, an important process that amplifies oxidative stress by generating H_2_O_2_ [98].

In mammalian cells, differences in gene expression between healthy cells and cancer cells result in DSR, which is the differential resistance of cancer cells to chemotherapeutics when combined with fasting [6, 13, 81]. In addition to DSR, fasting can also reduce cancer cell growth through DSS (Fig. 1, step 3). The impact of DSR and DSS appears to be mediated through different pathways, although the exact mechanisms are unclear. DSR is thought to be associated with the presence of oncogenic mutations in cancer cells, which cause cancer cells to bypass cell proliferation checkpoints and prevent these cancer cells from adapting to conditions of low nutrition [81, 99]. On the other hand, the extreme environment induced by fasting causes DSS, which makes cancer cells sensitive to chemotherapeutic drugs.

The outcomes of DSS is widely observed in cellular and animal models (Table 2) [22]. Interestingly, the benefits of DSS were most apparent when fasting is combined with chemotherapy, as both processes elevate the patient’s stress response. One study reported that when using CP drug (6–12 mg/ml) for ten hours, an increase of stress resistance was observed in six different rat and human glioma and neuroblastoma cancer cell lines under STF with low serum or glucose conditions [81]. STF can also protect healthy cells from chemotherapy, but not cancer cells. Low glucose and serum conditions have even increased the toxicity of chemotherapeutic drugs to cancer cells [81].

In addition to DSR and DSS mechanisms, fasting also reduced the expression of IGF-I. IGF-I protects cells from oxidative-stress-induced DNA damage [54]. Low levels of IGF-I can reduce mitogenic-related and apoptosis-related signaling pathways, including those regulated by Ras and Akt (Fig. 1, step 3). Mitosis is a necessary process for cell growth [100].

#### Strategy on treating metabolic syndrome during chemotherapy

Metabolic syndrome, including obesity and hyperglycemia, has been reported to be positively related to the risk of breast cancer and prostate cancer, and adversely affect the recurrence and prognosis of breast cancer [101, 102]. Moreover, research has shown that certain chemotherapy drugs, such as mTOR inhibitors and TKIs, can significantly alter lipid and glucose metabolism in the body [103]. For instance, mTOR inhibitors can increase plasma triglycerides and low-density lipoprotein (LDL) cholesterol by inhibiting lipoprotein lipase activity which may result in hyperlipidemia [103].

Besides, both L-asparaginase and glucocorticoid-based chemotherapy can cause hyperglycemia [104]. Clinical studies have shown that obesity makes female patients more susceptible to the toxicity of anticancer drugs [105]. We previously discussed the effect of fasting on reducing glucose levels (see The First Physiological Changes upon Fasting), which has been confirmed by the results of blood tests in healthy men 72 h after fasting [106]. Type 2 diabetes is a common comorbidity of breast cancer and can be diagnosed with glycated hemoglobin (HbA_1__C_) levels [107]. The level of HbA_1__C_ is positively correlated with cancer incidence. A study of early breast cancer found that an increase in fasting time at night was associated with lower HbA_1c_ levels and that fasting for more than 13 h during sleep had a positive effect on cancer and metabolic processes [108, 109]. Lipid metabolism is also regulated under fasting conditions. Compared with a non-fasting state, the lipoproteins remaining after fasting for more than 8 h are only of liver origin. During the fasting process, the energy supply of triglycerides was significantly reduced through lipolysis and oxidation [110], which will be most significant after fasting of 18–24 h [106]. Therefore, although there is no clinical evidence that fasting can treat or release metabolic complications during chemotherapy, fasting can provide strategies to help patients overcome the negative effects of metabolic syndrome.

#### Influence on EMT phenotypes

Metastatic cancer is associated with increased patient mortality [111]. The presence of metastases is intertwined with the process of epithelial-to-mesenchymal transition (EMT). Triggering the EMT pathways increases the overall motility of cells [112] and promotes cancer invasion and metastasis [113]. To evaluate the EMT status, the expression levels of E-cadherin or other proteins associated with cell-cell adhesion, such as vimentin and catenin, are usually quantified [113, 114].

The impact of nutrients on cancer through the EMT pathways has long been postulated, but few studies demonstrated the exact mechanisms involved. Recent studies based on cell and animal models have shed light on this association. In a recent study, gemcitabine, in combination with fasting simulation medium (FMM), significantly reduced cell migration in pancreatic cancer cells. FMM consists of DMEM containing 0.5 g/L glucose and 1% FBS. It is worth noting that FMM alone is as effective as a combination therapy that inhibits cell migration [9]. In other studies, asparagine levels were suggested to promote the formation of breast circulating tumor cells (CTCs) and enhance metastasis through EMT related proteins such as Twist1 and E-cadherin [115]. CTCs are a subset of cancer cells shed from tumors into the peripheral bloodstream and are believed to play a pivotal role in initiating metastasis [116]. Glucose levels are also considered to be factors that induce endometrial cancer cell proliferation and renal tubular epithelial cell EMT progression through the MAPK signaling pathway [117, 118].

IGF-1 is associated with enhanced tumor cell division and anti-apoptosis behaviour (see Nutrient Levels Influence Mechanisms that Promote Cancer Development) [55]. Studies have shown that an IGF-1 receptor, termed as insulin-like growth factor receptor 1 (IGF-R1), could induce phosphorylation of EMT markers such as β-catenin [119, 120]. IGF-R1 could also cause E-cadherin dissociation, leading to weaker cell-cell adhesions [121]. Overall, fasting strategies have significant impacts on tumor invasion and metastasis by inhibiting the EMT pathways [122].

### Potential caveats of implementing fasting strategies

#### Cancer stages and cancer types

Currently, no studies have compared the effects of fasting on patients at different stages and types of cancer, due to influences of metabolic changes with disease progression.

As far as cancer staging is concerned, fasting has been demonstrated in early and advanced patients with various types of cancer, including ovarian or uterine cancer. For example, patients with early-stage ovarian cancer fasted before and 24 h after chemotherapy and reported improved whole blood counts [8]. Side effects of chemotherapy in patients with advanced uterine cancer can be reduced through a fast for 36 h before chemotherapy, without affecting the efficacy of chemotherapy. Studies involving both early and advanced cancer types have shown that fasting can be safely used as an adjunct to chemotherapy and does not induce weight loss [28].

According to currently reported studies, fasting seems to treat a variety of cancers. To date, the types of cancer in clinical patients who have fasted and effectively treated include breast cancer, prostate cancer, esophageal adenocarcinoma, ovarian cancer, lung cancer, and uterine cancer [8, 27–30]. This degree of non-specificity maybe because the effects of fasting are mainly due to differences in metabolic factors between healthy cells and cancer cells, such as the Warburg effect (see Cancer Prevention and Treatment Enhancement) DSS and DSR (see Protective Effect against Chemotoxicity and Resistance and Stress Response).

Metabolic complications associated with the onset of cancer may affect fasting efficacy. For example, cachexia is a complex syndrome characterized by weight loss, reduced food intake, and systemic inflammation [123]. Weight loss is associated with acute-phase protein expression, such as elevated C-reactive protein and fibrinogen levels and decreased albumin levels [124]. Cancer cachexia syndrome is widespread in patients with cancer, and there is currently no specific effective treatment [125]. Studies on patients with cachexia pancreatic cancer have shown that protein synthesis during the acute phase increases during feeding as compared to fasting [124]. Fat metabolism consumption is another feature of malignant tumor cachexia [126]. However, another study showed that patients with fasting tumors have greater lipolysis than patients with stable weight [126]. A common dietary recommendation is to eat low-fat snacks regularly and take oral supplements [125, 127]. It is also assumed that patients who are susceptible to or fasting on a fasting diet are prone to malnutrition, sarcopenia, and cachexia risk. Still, no such cases have been reported [128]. Hence, fasting may not be suitable for cachexia patients.

Overall, studies involving early and advanced cancer types have shown that fasting can be safely used as an adjunct to chemotherapy and does not cause weight loss [28]. It will be useful to guide future research on fasting to study the effects on cancer cells under various metabolic conditions.

#### Heterogeneity in the tumor microenvironment

Cancer heterogeneity is a factor that leads to differences in the expression levels of metabolic-related genes in cancer [129]. Studies have shown that due to the activity of various metabolic pathways used for tumor growth and reproduction in tissues, certain normal metabolic functions are impeded, resulting in reduced levels of metabolic gene expression in digestive system tumors (e.g., colon, liver).

The tumor microenvironment has been widely demonstrated to induce disease heterogeneity and enhance tumor progression [130]. Cell heterogeneity is a caveat for the design of efficient treatments, as the different subpopulations of a tumor may react differently to drugs. There are two types of tumor heterogeneity, intra-tumor heterogeneity (within a tumor) and inter-tumor heterogeneity (across different tumors). This heterogeneity is also observed in cancer cells shed from the tumors [131, 132].

In other types of tumors, such as the cervix, the expression of metabolic genes is up-regulated. Metabolic heterogeneity exists between tumors of different subtypes in the same organ. Compared to areas with less perfusion, areas with higher lung tumor perfusion showed a greater tendency to use non-glucose nutrients (non-glucose carbon sources such as fatty acids, lactic acid, ketones) as energy sources [133].

However, current research on fasting in cancer treatment does not mention the effect of cancer heterogeneity on fasting efficacy. Cell lines and animal models are currently widely used to study the effects of fasting. Compared to other mammals, dog and mouse models are the most commonly used due to their relatively small size and ease of handling. With the exploration and clinical research of fasting treatment strategies to study its impact on chemotherapy and reveal possible side effects, cancer heterogeneity will become a key consideration. There is a high need for clinically relevant tumor models to accurately define the boundaries and parameters of fasting to enhance chemotherapy and further manage patient treatment strategies.

## Discussion and outlook - future considerations for personalised treatment

Due to the limited research available, we still cannot conclude that fasting can be widely used clinically. However, the potential prospects for implementing these fasting strategies with current and new treatment strategies remains exciting. For example, fasting could also be beneficial for enhancing cancer immunotherapy strategies. In cancer immunotherapy, cancer-specific T cell activation is induced to enhance the killing effect on cancer cells [134]. However, immunosuppression induced by chemotherapy can reduce the effectiveness of immunotherapy [17]. Studies have shown that conventional fasting techniques could alleviate the onset of chemotherapy-induced immunosuppression, reduce treatment-related mortality, and facilitate the protection and self-renewal of hematopoietic stem cells (HSCs) [135].

In the following sections, we conclude by providing recommendations for potential fasting techniques that can replace conventional STF.

### Fasting-mimicking diet (FMD) for Cancer immunotherapy

FMD is an emerging strategy with fewer side effects (Table 1). FMD retains the advantages of conventional fasting methods, such as a reduction in IGF-1 levels [16]. Although STF involves removing all nutrient intake, the FMD diet reduces calorie, protein, and sugar intake [16]. Studies have shown that chemotherapy, in combination with FMD, can enhance therapeutic effects, in the case of both conventional chemotherapy and immune-based cancer therapies. These effects were mediated by an increase in lymphoid progenitor cells and tumor-infiltrating lymphocytes (TILs) [17]. By reducing the expression of heme oxygenase-1 (HO-1), the downregulation of regulatory T cells, and the increase in CD8^+^ TIL-dependent cytotoxicity also affect the prognosis, which is a stress-reactive enzyme [17, 136]. Studies demonstrated that three cycles of 5-day FMD have a greater impact on high-risk factors or metabolic indicators (such as high body mass index (BMI), blood pressure, fasting blood glucose, triglycerides, C-reactive protein (CRP), cholesterol, and IGF) than those at physiological levels [137].

### Periodic fasting and Refeeding cycles (PFRC)

Periodic fasting or refeeding cycles (PFRC) is a set of systematic and regulated dietary regimens to improve the overall health status via control of the amount and time of energy intake (Table 1) [18]. Compared with traditional fasting techniques, PFRC improves overall tolerance to fasting-induced stress in four months and has a higher survival rate [18]. In preliminary studies using animal models, PFRC also showed higher efficacy in prolonging overall survival compared to *ad libitum* (AL) techniques [18]. Unlike STF, AL techniques involve free access to purified diet and drinking water (Table 1). Similar observations were recorded in animal models transplanted with lung cancer, liver cancer, or ovarian cancer cell lines under PFRC treatment, such as reduced tumor proliferation and regression [18]. Interestingly, studies involving PFRC strategies reported no toxicity effects, such as weight loss or DNA damage. However, other studies were conflicting and suggested that PFRC induce abnormally high cellular proliferation, which leads to increased canceration and precancerous lesions in tissues including liver and colon [138].

## Conclusions

STF can have a significant effect on the design of anti-cancer treatment strategies, primarily when used in combination with chemotherapy. However, the implementation of fasting strategies remains a challenge for patients due to the need for specialised diets or drugs [13].Currently, there are no randomised controlled trials that can conclude the clinical efficacy of fasting on humans. Existing research has focused on parameters such as reducing chemotherapy-related side effects [8, 27, 28] or implementation safety [30]. To achieve clinical utility, especially in the area of personalised treatment, follow-up studies and larger cohorts to study overall survival or disease-free survival parameters for clinical use is required. These studies will have to focus on the impact of fasting on extended periods, reveal mechanisms related to the benefits of fasting, and identify patient subtypes that are most suitable for fasting strategies. Studies should also target the management of personalized fasting strategies based on the individual physical condition of each patient and tailor each treatment based on factors such as cancer type and treatment regime to realise clinical utility. Overall, future research should target the development of clinically relevant tumor models that can accurately define the boundaries and parameters of fasting to enhance treatment strategies for chemotherapy and further treatment of patients.

## Data Availability

Not Applicable.

## Abbreviations

AA: Amino acids
AC: Adenylate cyclase
Akt: Protein kinase B
AL: *Ad libitum*
AMPK: AMP-activated protein kinase
Bad: Bcl2-associated death promoter
BCL2: B-cell lymphoma 2
BMI: Body mass index
*C. elegans*: *Caenorhabditis elegans*
cAMP: Cyclic adenosine monophosphate
CH_3_Se^−^: Methylselenol
CP: Cyclophosphamide
CREB: cAMP response element-binding protein
CRP: C-reactive protein
CTCs: Circulating tumor cells
DHA: Dehydroascorbic acid
DR: Dietary restriction
DSS: Differential stress sensitization
DSR: Differential stress resistance
DXR: Doxorubicin
*E. coli*: *Escherichia coli*
*Eef1g*: Elongation factor 1γ
EMT: Epithelial-to-mesenchymal transition
EPI: Epinephrine
FA: Fatty acid
FMD: Fasting-mimicking diet
FMM: Fasting simulation medium
GAP: Glycerol-phosphoric acid
GH: Growth hormone
GHR: Growth hormone receptor
GLU: Glucose
Gly: Glycerol
GPx: Glutathione peroxidase
Grx: Glutaredoxin
GSH: Glutathione
GSK-3β: Glycogen synthase kinase 3 beta
H_2_O_2_: Hydrogen peroxide
HER2: Epidermal growth factor receptor 2
HO-1: Heme oxygenase-1
HSC: Hematopoietic stem cell
HSe^−^: Hydrogen selenide
HSP: Heat shock protein
IF: Intermittent fasting
IGF-1: Insulin-like growth factor-1
IGF-R1: Insulin-like growth factor receptor 1
IGFBP: Insulin-like growth factor binding protein
IGFBP-3: Insulin-like growth factor binding protein-3
IR: Insulin receptor
Irs2: Insulin signaling adaptor
LDL: Low-density lipoprotein
Leu: Leucine
MAP: Microtubule-associated protein
MAPK: Mitogen-activated protein kinase
MET: Methionine
MR: Methionine restriction
mTOR: Mammal target of rapamycin
mTORC-1/2: Mammal target of rapamycin complexes 1/2
NAD^+^/NADH: Oxidized and reduced form of nicotinamide adenine dinucleotide
NK: Natural killer
O_2_^−^: Superoxide
PBMCs: Peripheral blood mononuclear cells
PEP: Phosphoenolpyruvate acid
PF: Periodic fasting
PFRC: Periodic fasting and refeeding cycles
PI3K: Phosphatidylinositol-3-kinase
PKA: Protein kinase A
PTEN: Phosphatase and tensin homolog
PUFA: Polyunsaturated fatty acid
ROS: Reactive oxygen species
*S. cerevisiae*: *Saccharomyces cerevisiae*
SIRT1: Sirtuin 1
STF: Short-term fasting
TAC: Docetaxel/doxorubicin/cyclophosphamide
TCA: The carboxylic acid
TILs: Tumor-infiltrating lymphocytes
TKI: Tyrosine kinase inhibitors
TNF: Tumor necrosis factor
TOR: Target of rapamycin
TRAIL: TNF-related apoptosis-inducing ligand
Trp: Tryptophan
Trx: Thioredoxin
TSC2: Tuberous Sclerosis Complex 2
UbQ: Ubiquinone
α-TOS: Alpha-tocopheryl succinate
γ/δ-TT: γ- tocotrienols/ δ-tocotrienols
